# Bias and Efficiency Comparison Between Multiple Imputation and Available-Case Analysis for Missing Data in Longitudinal Models

**DOI:** 10.1007/s12561-025-09493-6

**Published:** 2025-06-12

**Authors:** Panpan Zhang, Sharon X. Xie

**Affiliations:** Department of Biostatistics, Epidemiology and Informatics, University of Pennsylvania, 423 Gaurdian Drive, Philadelphia, PA 19104 USA

**Keywords:** Available-case analysis, Bias, Efficiency, Linear mixed-effects model, Missing data, Multiple imputation

## Abstract

In this paper, we compare the performance of available-case analysis (ACA) and several multiple imputation (MI) approaches for handling missing data problems in longitudinal analysis through estimation bias and relative efficiency. When the missingness of covariates depends on observed responses, ACA produces estimation bias, but it is preferred when there are only missing values in longitudinal responses. Multilevel MI methods are not always a solution to longitudinal data analysis. Single-level MI methods, like fully conditional specification (FCS), provide unbiased estimates under a variety of missing data scenarios, and improve efficiency gain in certain scenarios. The general assumption of missing data mechanism is missing at random (MAR). We carry out a systematic synthetic data analysis where missing data exist in longitudinal outcomes or/and covariates under different kinds of missing data generation procedures. The analysis model is a linear mixed-effects model. For each of the missing data scenarios, we give our recommendation (between ACA and a specific MI method) based on theoretical justifications and extensive simulations. In addition, a longitudinal neurodegenerative disease dataset is used as a real case study.

## Introduction

In longitudinal studies, each observational unit in the cohort is measured at baseline and repeatedly over time. Missing data are common in longitudinal analysis [1]. Various reasons may cause missing data, such as excessive duration of trials, invasive biomarker testing, high cost of therapies, among others. Missing data can occur in response variables (e.g., cerebrospinal fluid tau and beta amyloid plagues in Alzheimer’s disease [2]), time-varying covariates measured at each of the subsequent time points (e.g., gestational weight gains [3]) and fixed covariates measured only at baseline (e.g., APOE genotype [2]). For longitudinal observations and time-varying covariates, the pattern of missing data does not have to be monotone [4]; for instance, a participant may be absent at one follow-up visit, but returns to the study afterward. Missing data usually introduce estimation bias and cause loss of efficiency in statistical inference for longitudinal analysis.

Missing data are, in general, categorized into three mechanisms [5]: missing completely at random (MCAR), missing at random (MAR), and missing not at random (MNAR). By convention, MCAR and MAR are both considered as mild regularity assumptions in likelihood and Bayesian paradigms, but in practice, MAR appears more realistic than MCAR. The simplest way of handling missing data is complete-case analysis (CCA), which confines to the cases with no missing values. CCA bases inference only on completely observed cases, so accordingly undergoes the loss of information, inducing two main limitations: emergence of bias and loss of precision [4]. Notice that CCA filters out the cases containing partial information. An alternative procedure that includes all cases where the variable of interest is present, is termed available-case analysis (ACA). ACA outperforms CCA in a variety of situations as it utilizes more information contained in observed data, but usually gives severely misleading inference when the variables with missing data are highly correlated [6].

The limitations of CCA and ACA motivate the development of advanced methods that can better handle missing data in statistical analysis, such as maximum likelihood estimation (MLE), multiple imputation (MI), fully Bayesian approach and weighted estimation equations [7, 8]. To date, many of these approaches are considered standard methods. In this paper, we focus on MI [9], the fundamental idea of which is to substitute missing data by plausibly suitable values via a parametric model or a nonparametric procedure, hence allowing for complete data analysis on imputed datasets.

Since MI was first introduced to the community by Rubin [9], it has become a widespread method in both academia and industry owing to simplicity and versatility. Meng [10] discussed the issue of inference with uncongenial sources of data input when using MI; Schafer and Olsen [11] demonstrated the key ideas and software implementation of MI; Murray [12] provided a critical review about theoretical finds of MI. Nevertheless, a practical question that remains substantial interest is whether or not MI is always a better choice than CCA (or ACA)? Most of the classical studies were focused on missing values in response variables only until an investigation of missing covariates in simple linear regressions conducted by [6] in the 1990 s. More recently, a number of simulation-based studies were carried out for comparing MI and CCA in linear regression models [13, 14] and logistic regression models [15]. It is evident that, for linear regressions, the selection between CCA and MI should not only depend on the proportion of missing data [16]. Recently, Carpenter and Smuk [17] provided a critical and insightful review of this collection of studies.

In another recent study, Cao et al. [18] compared a series of MI methods (but not with ACA) with missing data existing in (univariate or multivariate) longitudinal outcomes only. The goal of our research is to comprehensively assess and compare the performances of MI and ACA under the longitudinal setting by considering missing data in a variety of missing data scenarios: missing data in covariates, responses, or both. Three major classes of MI approaches are included in our investigation: standard (single-level) MI, multilevel MI, and nonparametric MI. We would like to clarify that this paper mainly focuses on traditional MI methods, although with the booming development of machine learning techniques, especially neural networks, advanced deep learning (DL) algorithms have recently been applied to missing data imputation [19, 20]. Following the suggestion of one anonymous reviewer, we briefly investigate a specific deep learning algorithm in some of the simulations as a preliminary study but do not conduct a comprehensive, systematic exploration since it is beyond the scope of this paper.

MAR is assumed throughout the investigation, and only univariate outcomes are accounted. The analysis model considered is a linear mixed-effects model. Both probabilistic jurisdictions and extensive simulations are provided in support of our conclusions and recommendations [21]. The synthetic data for simulations are generated according to Parkinson’s Progression Markers Initiative (PPMI) data, the estimation results of which will also be presented as a case study.

The remainder of the manuscript is organized as follows. We briefly introduce the missing data mechanisms, and the notations that will be used throughout the manuscript in Sect. 2. In Sect. 3, we give a concise review of the three classes of MI methods. The longitudinal model for the analysis is set up in Sect. 4, with some probabilistic jurisdictions. An extensive simulation study and a real data analysis are, respectively, presented in Sects. 5 and 6. Lastly, we summarize the research outcomes and address some concluding remarks in Sect. 7.

## Missing Data Scenarios

There are different kinds of reasons that may cause the emergence of missing data. The nature of the missing data mechanism helps guide the decision on appropriate missing data strategies, varying from simple CCA to imputation-based methods. For $i = 1,2,\ldots ,n$, consider a dataset that consists of responses $\boldsymbol{Y}_i = (Y_{i1}, Y_{i2}, \ldots , Y_{i n_{i}})^\textsf{T}$, where $n_i$ is the number of repeated measures for subject *i*. Let $\boldsymbol{X}_i = (\boldsymbol{X}_{i1}, \boldsymbol{X}_{i2}, \ldots , \boldsymbol{X}_{i n_{i}})^\textsf{T}$ denote the corresponding covariate matrix, where each $\boldsymbol{X}_{ij}$, $j = 1, 2, \ldots , n_i$, has dimension $p \ge 1$.

Standard longitudinal analysis tools, such as mixed-effects models [22], use complete data and the data of subjects with outcomes missing at some time points, but have to remove the data with missing covariates. In this article, we consider missing data that concurrently exist in responses and/or covariates. Specifically, our goal is to assess and compare the performances of MI and ACA when missing data exist in responses only, covariates only, or both under various missing data generation procedures. Let us define two missing data indicators $\boldsymbol{R}_i = (\boldsymbol{R}_{i1}, \boldsymbol{R}_{i2}, \ldots , \boldsymbol{R}_{i n_{i}})^\textsf{T}$ and $\boldsymbol{S}_i = (S_{i1}, S_{i2}, \ldots , S_{i n_{i}})^\textsf{T}$ for $\boldsymbol{X}_i$ and $\boldsymbol{Y}_i$, respectively. More precisely, we set $R_{ijq} = 1$ if $X_{ijq}$, for $q = 1, 2, \ldots , p$, is observed and $S_{ij} = 1$ if $Y_{ij}$ is observed; 0, otherwise.

### Missing Data Mechanisms

As mentioned previously, there are three primary missing data mechanisms [5]: MCAR, MAR, and MNAR, where the term MCAR indicates that the missingness is unrelated to the data, rendering that the collection of complete cases becomes a random sample of the complete dataset, and hence a representative sample of the target population. Let $\boldsymbol{\psi }$ denote a collection of parameters governing the model for the emergence of missing data. Under MCAR, we have ${\textbf {Pr}}(\boldsymbol{R}_i, \boldsymbol{S}_i \mid \boldsymbol{X}_i, \boldsymbol{Y}_i;\boldsymbol{\psi }) = {\textbf {Pr}}(\boldsymbol{R}_i, \boldsymbol{S}_i;\boldsymbol{\psi })$, suggesting that the inference based on CCA or ACA is reliable and accurate, and consequently there is no bias in the estimates [4]. However, the loss of precision usually arises owing to the reduction in sample size. Little’s test [23] is a standard approach for testing MCAR. In most practical applications, MCAR does not seem to be a realistic missing data assumption. In contrast, a more conventional missing data assumption is MAR, i.e., the cause of missing values does not depend on unobserved data, given the observed data. That is ${\textbf {Pr}}(\boldsymbol{R}_i, \boldsymbol{S}_i \mid \boldsymbol{X}_i, \boldsymbol{Y}_i;\boldsymbol{\psi }) = {\textbf {Pr}}(\boldsymbol{R}_i, \boldsymbol{S}_i \mid \boldsymbol{X}_{i,{{\text {obs}}}}, \boldsymbol{Y}_{i,{{\text {obs}}}};\boldsymbol{\psi })$, where $\boldsymbol{Y}_{i,{{\text {obs}}}}$ and $\boldsymbol{X}_{i,{{\text {obs}}}}$ are observed responses and covariates, respectively. In the literature, both of MCAR and MAR are considered to be ignorable [4]. See a thorough discussion of distinguishing MCAR and MAR for Bayesian and likelihood-based inferences [24]. Under the assumption of MAR, complete cases no longer constitute a random sample of the population. Accordingly, CCA and ACA may produce biased estimates [6], and lead to invalid inference.

Missing data are called MNAR if the missingness depends on the values of observed data as well as the values that would have been observed [4]. The goal of the present paper is to compare the performances of ACA and MI, where standard MI method programs are developed under the assumption of MAR. We hence assume MAR throughout the manuscript.

## Multiple Imputation

Multiple imputation [9] is one of the most widely used methods for solving missing data problems. Different from single imputation, MI draws more than one imputed values from the predictive distribution of the missing data to reflect uncertainty. Suppose that a dataset *T* can be partitioned into observed values $T_{{\text {obs}}}$ and missing values $T_{{\text {mis}}}$. Let *Q* be an estimand. The posterior distribution of $(Q \mid T_{{\text {obs}}})$ is given by

$${\textbf {Pr}}(Q \mid T_{{\text {obs}}}) = \int {\textbf {Pr}}(Q \mid T_{{\text {mis}}}, T_{{\text {obs}}}) {\textbf {Pr}}(T_{{\text {mis}}} \mid T_{{\text {obs}}}) \, {{\text {d}}} T_{{\text {mis}}},$$

where ${\textbf {Pr}}(T_{{\text {mis}}} \mid T_{{\text {obs}}})$ is a specified imputation model, from which we can draw imputed values for $T_{{\text {mis}}}$, denoted by $\dot{T}_{{\text {mis}}}$. In what follows, we are able to calculate the corresponding quantities of $\dot{Q}$ through the imputed datasets $(T_{{\text {obs}}}, \dot{T}_{\text {n}is})$ containing no missing value. A sufficiently large collection of $\dot{Q}$ well approximates the posterior distribution of *Q* given the observed values in the original dataset. By applying the law of large numbers and the law of total variance, we are able to derive an unbiased and consistent estimator of *Q* as well as a variance estimator that simultaneously accounts for within-imputation and between-imputation variations, known as the Rubin’s rule [9].

There is a set of conditions required for MI to yield proper imputation and accordingly valid inference [9]. However, these proper procedures are difficult to check in practice [25]. Alternatively, several recent research summarized a list of specific circumstances, under which CCA is valid for regression models [13, 25]. It is worthy of noting that the inference by many modern MI methods is rooted in the Bayesian framework. However, the underlying theory of MI is not our primary concern in the present article. We refer the interested readers to several text-style expositions [4, 9, 26] for details. In the next two subsections, we look into several classes of popular MI approaches.

### Single-level Parametric MI

Single-level parametric MI procedures are model-based, where the two most well-known approaches are joint modeling (JM) and fully conditional specification (FCS). JM assumes a multivariate distribution for the entire dataset. Imputations for missing data are drawn from the fitted distribution. Multivariate normal (MVN) is the most widely accepted choice for both continuous and categorical data since the conditional marginal distributions of the random variables jointly following MVN can be fully specified. A comprehensive derivation and the underlying theoretical foundation of JM can be found in Little and Rubin [26], Schafer [4].

While the assumption of multivariate normality is severely violated, one considers an alternative, FCS [27, 28], where the imputation procedure is implemented based on one variable after another. The complexity of FCS is reflected in the demanding of the specification of an imputation model for each of the variables containing missing values. However, the method grants more flexibility to the imputation procedure. The imputation process is usually done in an iterative manner, for instance, the Gibbs sampling [29]. FCS can be regarded as a combination of several univariate imputations, so it may undergo an issue of compatibility [30]. Besides, since the algorithm of FCS is based on Markov chain Monte Carlo (MCMC) methods, the assessment of convergence can be another concern [31].

Under the assumption of MVN, JM is identical to FCS [32]. In the present study, we implement this class of imputations by exploiting the built-in functions from mice package, which is available on the CRAN. A variety of syntax examples and associated demonstrations are given in the tutorial by Van Buuren and Groothuis-Oudshoorn [33].

### Multilevel MI

Longitudinal data can be viewed as multilevel data. Multilevel MI was originally developed for complex data with hierarchical or clustered structure, and now has been extended to longitudinal data analysis. Missing data in longitudinal studies can occur in repeated outcomes, time-varying covariates or fixed covariates, respectively, corresponding to outcome variables, level-one predictors and level-two predictors in the context of multilevel MI. The primary difference between multilevel and single-level MI methods is that the former accounts for random effects in the imputation procedure. Like single-level MI, multilevel MI is also done in a Bayesian framework [34]. For instance, in a random intercepts model, multilevel MI introduces a new variable modeling random intercepts in addition to the others containing missing values in the imputation procedure. Under such setting, the estimates of the random intercepts are hence updated at each iterative step, alongside the others for regression coefficients. Consequently, the algorithms for multilevel MI, e.g., MCMC-based method, usually demand more iterations to converge, where the rate of convergence depends on the magnitude of the correlation between iteration steps [35].

In mice, several imputation methods have been developed for multilevel models [34, 36]. Though it is evident that using single-level imputation methods in multilevel models may underestimate intra-class correlations [37], the effectiveness of multilevel MI is related to the type of variables containing missing values in the model. Most existing multilevel MI methods are well developed for dealing with the missing values that occur in longitudinal outcomes, but not necessarily for the problems with missing data in covariates [25]. Besides, a few practical issues arising from the nature of longitudinal data may have caused more challenges in statistical analysis; for instance, small number of follow-ups, insufficient number of subjects in the cohort, or missing data following some sort of systematical pattern. Multilevel MI is not yet completely developed, so requires further research forces.

### Nonparametric MI

In a large body of real data analyses, neither analysis model nor imputation model is known. Moreover, the relations of responses and covariates are not always linear, and the assumption of MVN often remains questionable, too. When the knowledge about the true relationships among the variables of interest is extremely limited, using model-based imputation approaches may lead to severely misleading results. One proposes a machine-learning-based alternative to drawing imputations for missing data, called Classification and Regression Trees [CART, 38]. The fundamental idea of the CART algorithm is to recursively partition the predictor space such that the ultimate units are formed by relatively homogeneous outcomes [39], where the partitioning criteria bring out the sampling pools of imputations.

It is evident that nonparametric MI methods, like CART, can provide more reliable inference in complex models [38]. However, statisticians sometimes still give preference to model-based methods in practice since most of the nonparametric (or semi-parameteric) MI approaches are lack of theoretical foundation [12, 40]. In this article, we select CART as a competing method, for the purpose of determining and demonstrating the scenarios, for which nonparametric MI approaches should be considered.

## Model Setup

Mixed-effects models are a powerful and prominent tool for analyzing longitudinal data. We consider a linear mixed-effects model (LMM), also known as the Laird-Ware model [22], in the present study. The LMM, in general, is able to handle unbalanced data, i.e., the numbers of visits for the participants indexed with *i*, $n_i$, do not have to be identical. As data balance is not our main concern in this study, we assume $n_i = m$ for all *i* for the simulations in Sect. 5. Formally, the LMM is given by

$$\boldsymbol{Y}_{i} = \boldsymbol{X}_i\boldsymbol{\beta }+ \boldsymbol{Z}_{i}\boldsymbol{b}_{i} + \boldsymbol{\varepsilon }_{i},$$

where $\boldsymbol{Y}_i$ is an $m \times 1$ vector of observations, $\boldsymbol{X}_i$ is an $m \times p$ matrix of fixed-effects covariates, $\boldsymbol{\beta }$ is a *p*-dimensional vector of regression coefficients, $\boldsymbol{Z}_i$ is a known $m \times q$ design matrix linking $\boldsymbol{Y}_i$ and $\boldsymbol{b}_i$ which is a *q*-dimensional vector of random effects, and $\boldsymbol{\varepsilon }_i$ is an *m*-dimensional vector of error terms. The Laird-Ware model further assumes that

$$\boldsymbol{\varepsilon }_i \overset{{{\text {ind.}}}}{\sim } \mathcal{N}(0, \sigma ^2 \boldsymbol{I}) \quad \text{ and } \quad \boldsymbol{b}_i \overset{{{\text {i.i.d.}}}}{\sim } \mathcal{N}(0, \boldsymbol{D})$$

are mutually independent, where $\boldsymbol{I}$ is an $m \times m$ identity matrix and $\boldsymbol{D}$ is an unknown $q \times q$ variance-covariance matrix for random effects. Under these assumptions, one can show

$$(\boldsymbol{Y}_i \mid \boldsymbol{X}_i, \boldsymbol{Z}_i; \boldsymbol{\beta }, \sigma ^2, \boldsymbol{D}) \sim \mathcal{N}\left( \boldsymbol{X}_i \boldsymbol{\beta }, \boldsymbol{Z}_i \boldsymbol{D}\boldsymbol{Z}_i^\textsf{T} + \sigma ^2 \boldsymbol{I}\right) ,$$

giving rise to a likelihood-based estimation algorithm for LMM inference proposed by Laird and Ware [22]. More specifically, one can treat the random effects as missing values, and adopt the Expectation-Maximization (EM) algorithm [41] to estimate the target parameters and random effects simultaneously.

Under the assumption of MAR, it is evident that ACA (or CCA) provides unbiased estimates for linear regression models as long as the missing data depend only on fully observed covariates, but not on observed responses [4, 8, 42]. Let $\mu (\cdot )$ be a well-defined Lebesgue measure for continuous random variable or a counting measure for discrete random variable. In addition, let $\boldsymbol{\theta }$ denote the collection of the parameters to estimate. If missing values occur in longitudinal responses only, then, for each subject *i*, we have

$$\begin{aligned}&{\textbf {Pr}}(\boldsymbol{Y}_{{{\text {obs}}}, i}, \boldsymbol{S}_i \mid \boldsymbol{X}_i, \boldsymbol{Z}_i; \boldsymbol{\theta },\boldsymbol{\psi }) \\&\qquad = \int {\textbf {Pr}}(\boldsymbol{Y}_{{{\text {obs}}}, i}, \boldsymbol{Y}_{{{\text {mis}}}, i}, \boldsymbol{S}_i \mid \boldsymbol{X}_i, \boldsymbol{Z}_i; \boldsymbol{\theta }, \boldsymbol{\psi }) \, {{\text {d}}} \mu (\boldsymbol{Y}_{{{\text {mis}}}, i}) \\ &\qquad = \int {\textbf {Pr}}(\boldsymbol{S}_i \mid \boldsymbol{Y}_i, \boldsymbol{X}_i, \boldsymbol{Z}_i; \boldsymbol{\psi }) {\textbf {Pr}}(\boldsymbol{Y}_{{{\text {obs}}}, i}, \boldsymbol{Y}_{{{\text {mis}}}, i} \mid \boldsymbol{X}_i, \boldsymbol{Z}_i; \boldsymbol{\theta }) \, {{\text {d}}} \mu (\boldsymbol{Y}_{{{\text {mis}}}, i}). \end{aligned}$$

Since there is no missing value in $\boldsymbol{X}_i$ or $\boldsymbol{Z}_i$, then, under the assumption of MAR, we arrive at

$$\begin{aligned} {\textbf {Pr}}(\boldsymbol{Y}_{{{\text {obs}}}, i}, \boldsymbol{S}_i \mid \boldsymbol{X}_i, \boldsymbol{Z}_i; \boldsymbol{\theta },\boldsymbol{\psi })&= \int {\textbf {Pr}}(\boldsymbol{S}_i \mid \boldsymbol{Y}_{{{\text {obs}}}, i}, \boldsymbol{X}_i, \boldsymbol{Z}_i ; \boldsymbol{\psi }) {\textbf {Pr}}(\boldsymbol{Y}_{i} \mid \boldsymbol{X}_i, \boldsymbol{Z}_i; \boldsymbol{\theta }) \, {{\text {d}}} \mu (\boldsymbol{Y}_{{{\text {mis}}}, i}) \\ &= {\textbf {Pr}}(\boldsymbol{S}_i \mid \boldsymbol{Y}_{{{\text {obs}}}, i}, \boldsymbol{X}_i, \boldsymbol{Z}_i; \boldsymbol{\psi }) \int {\textbf {Pr}}(\boldsymbol{Y}_{i} \mid \boldsymbol{X}_i, \boldsymbol{Z}_i; \boldsymbol{\theta }) \, {{\text {d}}} \mu (\boldsymbol{Y}_{{{\text {mis}}}, i}) \\ &= {\textbf {Pr}}(\boldsymbol{S}_i \mid \boldsymbol{Y}_{{{\text {obs}}}, i}, \boldsymbol{X}_i, \boldsymbol{Z}_i; \boldsymbol{\psi }) {\textbf {Pr}}(\boldsymbol{Y}_{{{\text {obs}}}, i} \mid \boldsymbol{X}_i, \boldsymbol{Z}_i ; \boldsymbol{\theta }). \end{aligned}$$

The separability in the last expression indicates that ${\textbf {Pr}}(\boldsymbol{S}_i \mid \boldsymbol{Y}_{{{\text {obs}}}, i}, \boldsymbol{X}_i, \boldsymbol{Z}_i; \boldsymbol{\psi })$ is free of $\boldsymbol{\theta }$, hence making no contribution to the likelihood of $\boldsymbol{\theta }$. In what follows, there is no need to distinguish a model for generating missing data when deriving MLEs.

As mentioned, limited work accounting for missing covariates has been done in the literature, especially in longitudinal studies. To the best of our knowledge, there are only a few research articles investigating missing dropouts as well as missing time-varying covariates under the assumption of MNAR in LMM-based longitudinal analysis [43–45]. We are interested in the performance of different imputation methods for handling missing data in longitudinal responses and/or time-invariant covariates under the assumption of MAR. More precisely, we would like to give recommendations of appropriate (standard) methods in response to different missing data scenarios.

Suppose that there is no missing value in $\boldsymbol{Y}_i$, i.e., $\boldsymbol{Y}_{{{\text {obs}}}, i} = \boldsymbol{Y}_i$, for the moment. In addition, we assume that $\boldsymbol{Z}_i$ is also fully observed. For each *i*, the likelihood based on observed data is given by

$${\textbf {Pr}}(\boldsymbol{Y}_i, \boldsymbol{R}_i \mid \boldsymbol{X}_{{{\text {obs}}},i}, \boldsymbol{Z}_i; \boldsymbol{\theta },\boldsymbol{\psi }) = {\textbf {Pr}}(\boldsymbol{R}_i \mid \boldsymbol{Y}_i, \boldsymbol{X}_{{{\text {obs}}},i}, \boldsymbol{Z}_i; \boldsymbol{\psi }) {\textbf {Pr}}(\boldsymbol{Y}_i \mid \boldsymbol{X}_{{{\text {obs}}},i}, \boldsymbol{Z}_i; \boldsymbol{\theta }).$$

If the missingness of $\boldsymbol{X}_i$ is independent of $\boldsymbol{Y}_i$, then we get

$${\textbf {Pr}}(\boldsymbol{R}_i \mid \boldsymbol{Y}_i, \boldsymbol{X}_{{{\text {obs}}},i}, \boldsymbol{Z}_i;\boldsymbol{\psi }) = {\textbf {Pr}}(\boldsymbol{R}_i \mid \boldsymbol{X}_{{{\text {obs}}},i}, \boldsymbol{Z}_i;\boldsymbol{\psi }),$$

which makes no contribution to the likelihood of $\boldsymbol{\theta }$. This means that the likelihood of the full data is proportion to the likelihood of the observed data (analogous to the justification for simple regression models [46]). For the general case that allows for missing values in $\boldsymbol{Y}_i$ as well, the above arguments, under the assumption of MAR, can be extended effortlessly by introducing the missing value indicator for $\boldsymbol{Y}_i$, i.e., $\boldsymbol{S}_i$:

$$\begin{aligned}&{\textbf {Pr}}(\boldsymbol{Y}_{{{\text {obs}}},i}, \boldsymbol{S}_i , \boldsymbol{R}_i \mid \boldsymbol{X}_{{{\text {obs}}},i}, \boldsymbol{Z}_i ; \boldsymbol{\theta },\boldsymbol{\psi }) \\ &\qquad = \int {\textbf {Pr}}(\boldsymbol{Y}_{{{\text {obs}}}, i},\boldsymbol{Y}_{{{\text {mis}}}, i}, \boldsymbol{S}_i , \boldsymbol{R}_i \mid \boldsymbol{X}_{{{\text {obs}}},i}, \boldsymbol{Z}_i; \boldsymbol{\theta }, \boldsymbol{\psi }) \, {{\text {d}}} \mu (\boldsymbol{Y}_{{{\text {mis}}}, i}) \\ &\qquad = \int {\textbf {Pr}}(\boldsymbol{S}_i , \boldsymbol{R}_i \mid \boldsymbol{Y}_i , \boldsymbol{X}_{{{\text {obs}}},i}, \boldsymbol{Z}_i; \boldsymbol{\psi }) {\textbf {Pr}}(\boldsymbol{Y}_i \mid \boldsymbol{X}_{{{\text {obs}}},i}, \boldsymbol{Z}_i; \boldsymbol{\theta })\, {{\text {d}}} \mu (\boldsymbol{Y}_{{{\text {mis}}}, i}) \\ &\qquad = \int {\textbf {Pr}}(\boldsymbol{S}_i , \boldsymbol{R}_i \mid \boldsymbol{Y}_{{{\text {obs}}}, i} , \boldsymbol{X}_{{{\text {obs}}},i}, \boldsymbol{Z}_i; \boldsymbol{\psi }) {\textbf {Pr}}(\boldsymbol{Y}_i \mid \boldsymbol{X}_{{{\text {obs}}},i}, \boldsymbol{Z}_i; \boldsymbol{\theta })\, {{\text {d}}} \mu (\boldsymbol{Y}_{{{\text {mis}}}, i}) \\ &\qquad = {\textbf {Pr}}(\boldsymbol{S}_i , \boldsymbol{R}_i \mid \boldsymbol{Y}_{{{\text {obs}}}, i}, \boldsymbol{X}_{{{\text {obs}}},i}, \boldsymbol{Z}_i; \boldsymbol{\psi }) {\textbf {Pr}}(\boldsymbol{Y}_{{{\text {obs}}}, i} \mid \boldsymbol{X}_{{{\text {obs}}},i}, \boldsymbol{Z}_i ; \boldsymbol{\theta }), \end{aligned}$$

which implies that ACA gives valid inference provided that the missingness of covariates (i.e., $\boldsymbol{R}_i$) does not depend on $\boldsymbol{Y}_i$.

Nevertheless, ACA (or CCA for regression models) always undergoes the loss of efficiency in estimation. Suppose that the likelihood of the full model with parameters $(\boldsymbol{\theta }, \boldsymbol{\phi })$, where $\boldsymbol{\theta }$ refers to the collection of parameters of interest and $\boldsymbol{\phi }$ refers to the collection of auxiliary parameters that may or may not exist, can be expressed as

$$\mathcal {L}(\boldsymbol{\theta },\boldsymbol{\phi }\mid \boldsymbol{Y}, \boldsymbol{X}, \boldsymbol{Z}, \boldsymbol{R}, \boldsymbol{S}) = \mathcal {L}_{{\text {ACA}}}(\boldsymbol{\theta }\mid \boldsymbol{Y}_{{\text {obs}}}, \boldsymbol{X}_{{\text {obs}}}, \boldsymbol{Z}) \times \mathcal {L}_{\text {r}em}(\boldsymbol{\theta }, \boldsymbol{\phi }\mid \boldsymbol{Y}_{{\text {mis}}}, \boldsymbol{X}_{{\text {mis}}}, \boldsymbol{Z}).$$

The likelihood for the remaining part (the latter term on the right-hand side of the above equation) is not considered in the estimation procedure via ACA. Accordingly, the likelihood-based estimation by using ACA loses efficiency, provided that $\mathcal {L}_{{\text {rem}}}$ contains information about $\boldsymbol{\theta }$. In other words, ACA is efficient only if the observed data contain all the information about the parameters to be estimated.

MI is an alternative to missing value problems, and appropriate MI is expected to remedy ACA (or CCA) if ACA produces biased estimates. Besides, appropriate MI is expected to recover efficiency gains relative to ACA. Standard single-level and multilevel MI method for LMM are both based on MCMC algorithms [34], where the algorithm of multilevel MI is regarded as an extension of that of single-level MI. Without loss of generality, suppose that the missing values occur in $\boldsymbol{Y}$ only. The major steps of an iterative algorithm for multilevel MI are given as follows. Let $\boldsymbol{\theta }^{(0)}$ and $\boldsymbol{Y}_{{\text {mis}}}^{(0)}$ be the initial values. For iteration $t \ge 1$,

1. Sample random effects $\boldsymbol{b}_i^{(t)} \sim {\textbf {Pr}}(\boldsymbol{b}_i \mid \boldsymbol{Y}_{{\text {obs}}}, \boldsymbol{Y}_{{\text {mis}}}^{(t - 1)}, \boldsymbol{\theta }^{(t - 1)})$ independently for $i = 1, 2, \ldots , m$;
2. Update $$\boldsymbol{\theta }^{(t)} \sim {\textbf {Pr}}(\boldsymbol{\theta }\mid \boldsymbol{Y}_{{\text {obs}}}, \boldsymbol{Y}_{{\text {mis}}}^{(t - 1)}, \{\boldsymbol{b}_i^{(t)}: i = 1, 2, \ldots , m\})$$;
3. Draw $\boldsymbol{Y}_{{{\text {mis}}}, i}^{(t)} \sim {\textbf {Pr}}(Y_{{{\text {mis}}}, i} \mid \boldsymbol{Y}_{{\text {obs}}}, \boldsymbol{b}_i^{(t)}, \boldsymbol{\theta }^{(t)})$ independently for $i = 1, 2, \ldots , m$.

The above steps are repeated until convergence takes place. Single-level MI methods do not account for random effects $\boldsymbol{b}_i$. When missing data exist in $\boldsymbol{X}$, $\boldsymbol{Y}$, and $\boldsymbol{Z}$, the corresponding iterative estimation algorithm is similar to pseudo codes presented above, where an imputation model is specified for each incomplete variable [33].

## Simulations

In this section, we conduct an extensive simulation study to assess the performances of ACA, FCS (single-level MI), CART (nonparametric MI), and PAN (multilevel MI, specifically referred to two-level PAN) under a variety of missing data scenarios. In addition, we perform complete data analysis (CDA) using LMM fitted to the complete data (before generating any missing data) and use the CDA results as reference (gold standard). For each scenario under consideration, we recommend an appropriate method based on estimation bias and relative efficiency if no bias is observed. Specifically, we consider the following LMM that consists of random intercepts only:

$$Y_{ij} = \beta _0 + b_{i} + \beta _1 X_{1,ij} + \beta _2 X_{2,i} + \beta _3 X_{3,i} + \beta _4 X_{4, i} + \varepsilon _{ij},$$

where $Y_{ij}$ is the observation of subject $i = 1, 2, \ldots , n$ at time $j = 1, 2, \cdots , m$, $\boldsymbol{\beta }= (\beta _0, \beta _1, \beta _2, \beta _3, \beta _4)$ is a vector of regression coefficients, $X_{1,ij}$ is a time-varying covariate, $X_{2,i}$, $X_{3, i}$, and $X_{4,i}$ are (continuous or categorical) time-independent covariates that are measured at baseline, $b_i \sim \mathcal {N}(0, d^2)$ is a subject-level random intercept, and $\varepsilon _{ij} \sim \mathcal {N}(0, \sigma ^2)$ is an error term that is assumed to be independent of $b_i$. We set $n = 400$ for all simulations and $m = 5$ for all *i*. The true parameter values $\boldsymbol{\beta }= (\beta _0, \beta _1, \beta _2, \beta _3, \beta _4)$ are $(31, -0.45, -0.07, 0.56, 0.4)$, motivated by the longitudinal data for Parkinson’s disease (PD) in Sect. 6. The standard deviations of the random intercepts and the error terms are, respectively, set to $d = 1.6$ and $\sigma = 1.8$.

We assume no missing value in $X_{1,ij}$, $X_{3,i}$, and $X_{4,i}$ throughout the simulation analysis. The missing data of $Y_{ij}$ is not required to be monotonic. In real longitudinal studies, it is quite common to have missing values in $Y_{ij}$, but the corresponding $X_{1,ij}$ is observed for given *i* and *j*, depending on the nature of $Y_{ij}$ and $X_{1,ij}$. For instance, suppose that $Y_{ij}$’s are the results of an expensive test of invasive biomarker, and $X_{1,ij}$’s are follow-up times. Doctors and neurologists may not suggest a patient to take the test unless it is necessary, but the follow-up time is recorded automatically as long as the participant does not drop out.

There are three missing data scenarios that we consider in the simulation study:

1. Missing data in $Y_{ij}$ only;
2. Missing data in $X_{2,i}$ only;
3. Missing data in both $Y_{ij}$ and $X_{2,i}$.

For each missing data scenario, we generate a total of $R = 500$ datasets according to various missing data generation processes. We will explicitly give the details about those missing data generation processes in the subsequent sections. Although we do not set a fixed missing data proportion in our simulation analysis, we intentionally control the average between $35 \%$ and $70 \%$, accommodating the actual situations discovered in most longitudinal data.

For each simulation, we report the following results: percentage of bias (PB), standard deviation (SD), standard errors (SE), relative efficiency (RE), and coverage probability (CP). We adopt percentage of bias rather than absolute bias since it is more informative when bias is small in magnitude. We report both of the average of standard errors and the empirical standard deviation (of the estimates) to justify consistency of estimating standard error (reflected in similar values between SE and SD). We boldface the RE rows of recommended methods in Tables 1, 2, 3, and 4, and boldface the PB row of recommended methods in Table 5 (since FCS in that table is the only one producing unbiased estimates). When conducting MI, we apply a data-driven algorithm to approximate the number of imputed datasets (per imputation) according to a broadly accepted guidance [47]. Specifically for CART, the number of donors needs to be predetermined, where 10 is selected according to a general recommendation [25].

### MCAR

MCAR is thought of as a special case of MAR. Under the assumption of MCAR, it seems plausible that MI would perform better than ACA as the method preserves sample size by imputing missing values via a statistically proper procedure [48]. At the same time, opposite arguments exist in the literature positing that CCA could have smaller SE than MI for regression models [49].

We generate missing data in $\boldsymbol{Y}_i$, in $\boldsymbol{X}_i$ or in both under the assumption of MCAR with fixed proportion of missing. Specifically, we consider missing proportion, respectively, set to $20\%$ (slightly missing), $40\%$ (modestly missing), and $60\%$ (severely missing) to assess whether the performance of missing data method is related to missing proportion or not. We observe a consistent pattern across three different levels of missing proportion in our simulation results so as to drive a unified conclusion. For brevity, we present the results for $60\%$ missing data proportion only in Table 1.

**Table 1:** Simulation results under the assumption of MCAR, with missing proportion $60\%$

	$\boldsymbol{S}; \boldsymbol{\psi }$					$\boldsymbol{R}; \boldsymbol{\psi }$					$\boldsymbol{S}, \boldsymbol{R}; \boldsymbol{\psi }$				
	$\beta _0$	$\beta _1$	$\beta _2$	$\beta _3$	$\beta _4$	$\beta _0$	$\beta _1$	$\beta _2$	$\beta _3$	$\beta _4$	$\beta _0$	$\beta _1$	$\beta _2$	$\beta _3$	$\beta _4$
CDA (PB)	0.17	0.17	0.53	3.10	0.05	0.17	0.17	0.53	3.10	0.05	0.17	0.17	0.53	3.10	0.05
CDA (SD)	0.64	0.03	0.01	0.20	0.09	0.64	0.03	0.01	0.20	0.09	0.64	0.03	0.01	0.20	0.09
CDA (SE)	0.61	0.03	0.01	0.19	0.09	0.61	0.03	0.01	0.19	0.09	0.61	0.03	0.01	0.19	0.09
CDA (RE)	1.00	1.00	1.00	1.00	1.00	1.00	1.00	1.00	1.00	1.00	1.00	1.00	1.00	1.00	1.00
CDA (PC)	0.93	0.95	0.94	0.94	0.96	0.93	0.95	0.94	0.94	0.96	0.93	0.95	0.94	0.94	0.96
ACA (PB)	0.20	0.03	0.80	2.78	0.61	0.29	0.18	1.33	3.26	1.21	0.23	0.09	0.93	2.58	2.31
ACA (SD)	0.77	0.05	0.01	0.24	0.11	0.98	0.04	0.01	0.30	0.14	1.23	0.08	0.02	0.37	0.18
ACA (SE)	0.74	0.05	0.01	0.23	0.11	0.98	0.05	0.01	0.30	0.14	1.18	0.08	0.02	0.36	0.17
ACA (RE)	**1.21**	**1.75**	**1.20**	**1.20**	**1.20**	1.60	1.58	1.60	1.59	1.60	1.92	2.77	1.92	1.91	1.91
ACA (PC)	0.94	0.96	0.94	0.93	0.96	0.96	0.95	0.96	0.94	0.96	0.94	0.95	0.94	0.94	0.94
FCS (PB)	0.18	0.00	0.74	2.48	0.53	0.14	0.17	0.32	3.08	3.14	0.22	0.06	0.99	2.71	3.25
FCS (SD)	0.77	0.05	0.01	0.24	0.11	0.65	0.03	0.01	0.20	0.13	0.79	0.05	0.01	0.25	0.17
FCS (SE)	0.75	0.05	0.01	0.23	0.11	0.64	0.03	0.01	0.20	0.14	0.78	0.05	0.01	0.24	0.17
FCS (RE)	1.23	1.82	1.22	1.22	1.22	**1.04**	**1.00**	**1.05**	**1.04**	**1.52**	**1.27**	**1.82**	**1.27**	**1.26**	**1.84**
FCS (PC)	0.94	0.96	0.94	0.93	0.96	0.95	0.95	0.95	0.94	0.95	0.94	0.96	0.95	0.94	0.94
CART (PB)	0.63	0.03	9.20	25.23	10.52	0.11	0.17	0.25	5.06	5.45	0.75	0.09	10.41	27.29	11.42
CART (SD)	0.71	0.05	0.01	0.19	0.10	0.65	0.03	0.01	0.20	0.15	0.72	0.05	0.01	0.19	0.16
CART (SE)	0.71	0.05	0.01	0.22	0.10	0.62	0.03	0.01	0.19	0.10	0.73	0.05	0.01	0.22	0.15
CART (RE)	1.17	1.61	1.16	1.15	1.16	1.01	1.00	1.01	1.01	1.16	1.19	1.61	1.19	1.15	1.67
CART (PC)	0.93	0.92	0.92	0.93	0.95	0.94	0.95	0.94	0.93	0.82	0.92	0.92	0.90	0.91	0.93
PAN (PB)	0.20	0.03	0.82	2.87	0.73	0.17	0.17	0.49	3.20	59.70	0.23	0.04	0.96	3.01	66.71
PAN (SD)	0.77	0.05	0.01	0.24	0.11	0.65	0.03	0.01	0.20	0.06	0.78	0.05	0.01	0.24	0.06
PAN (SE)	0.74	0.05	0.01	0.23	0.11	0.63	0.03	0.01	0.19	0.12	0.76	0.05	0.01	0.23	0.12
PAN (RE)	1.21	1.76	1.20	1.20	1.20	1.03	1.00	1.03	1.03	1.32	1.24	1.76	1.23	1.23	1.37
PAN (PC)	0.94	0.96	0.94	0.94	0.95	0.94	0.95	0.94	0.95	0.46	0.95	0.96	0.95	0.94	0.36

Missing values occur in $\boldsymbol{Y}_i$, $\boldsymbol{X}_i$, or both (from left to right). True values are $\boldsymbol{\beta }= (31, -0.45, -0.07, 0.56, 0.4)$

According to the simulation results, we see that ACA, FCS, and PAN all provide unbiased estimates when missing data exist in longitudinal outcomes only. For CART, the estimates of regression coefficients $\beta _3$ and $\beta _4$ are severely biased, though the rest are relatively accurate. Thus, CART does not seem to be an appropriate solution. When there is no missing value in covariates, the REs of the estimates from ACA and PAN are almost identical, and slightly better than those from FCS, since no additional information is obtained by using MI but the SEs may increase owing to the additionally introduced uncertainty [50]. Thus, we give preference to ACA as it save more computation powers than PAN. However, if there are missing data in covariates or both covariates and longitudinal outcomes, some of the estimates obtained by PAN are biased, especially when both longitudinal outcomes and covariates contain missing values. This is also consistent with known conclusions by Van Buuren [25]. On the other hand, the estimates from ACA and FCS are unbiased, and FCS has higher efficiency gains than ACA. Thus, we are in favor of FCS for the latter two missing data scenarios.

In the next three sections, our simulations are carried out under a more realistic assumption of MAR.

### Missing Data in Responses Only

In longitudinal analysis, it is most frequent to observe missing value in repeated outcomes due to subject dropouts. In this section, we assume that there is no missing value in $\boldsymbol{X}_{2,i}$, whereas the missing data of $\boldsymbol{Y}_i$ are generated in the following three different ways.

The first case is that $\boldsymbol{Y}_{{{\text {mis}}}, i}$ depends only on the observed values of $\boldsymbol{Y}_i$. To implement, we generate the missing values at the baseline under MCAR. The generation of missing values in the follow-ups depends on the observed responses at previous time points in an iterative manner. The second case is that $\boldsymbol{Y}_{{{\text {mis}}}, i}$ (including baseline outcomes) depends only on covariates $\boldsymbol{X}_i$, which are fully observed. More precisely, we propose a logistic model to generate missing data:

1 $$\begin{aligned} \boldsymbol{S}_{ij} = \texttt { logit}(\gamma _{0,i} + \boldsymbol{\gamma }_{1,i} \boldsymbol{X}_{1,i} + \gamma _{2,i} \boldsymbol{X}_{2,i} + \gamma _{3,i} \boldsymbol{X}_{3,i} + \gamma _{4,i} \boldsymbol{X}_{4,i}), \end{aligned}$$

where $\gamma$’s are predetermined parameters. We select appropriate $\gamma$’s such that the average missing proportion is close to that of PPMI data. The third case is an integration of the first two cases, allowing for the missingness of $\boldsymbol{Y}_{{{\text {mis}}}, i}$ simultaneously depending on previously observed outcomes as well as completely observed covariates. The simulation results for the three cases are all presented in Table 2.

**Table 2:** Simulation results for missing values occurring in $\boldsymbol{Y}_i$ only under the assumption of MAR, where the missingness of $\boldsymbol{Y}_i$, respectively, depends on observed responses, fully observed covariates, or both (from left to right)

	$\boldsymbol{S}\mid \boldsymbol{Y}_{\text {obs}} \; (62\%)$					$\boldsymbol{S}\mid \boldsymbol{X}_{\text {obs}} \; (63\%)$					$\boldsymbol{S}\mid \boldsymbol{Y}_{\text {obs}}, \boldsymbol{X}_{\text {obs}} \; (61\%)$				
	$\beta _0$	$\beta _1$	$\beta _2$	$\beta _3$	$\beta _4$	$\beta _0$	$\beta _1$	$\beta _2$	$\beta _3$	$\beta _4$	$\beta _0$	$\beta _1$	$\beta _2$	$\beta _3$	$\beta _4$
CDA (PB)	0.17	0.17	0.53	3.10	0.05	0.17	0.17	0.53	3.10	0.05	0.17	0.17	0.53	3.10	0.05
CDA (SD)	0.64	0.03	0.01	0.20	0.09	0.64	0.03	0.01	0.20	0.09	0.64	0.03	0.01	0.20	0.09
CDA (SE)	0.61	0.03	0.01	0.19	0.09	0.61	0.03	0.01	0.19	0.09	0.61	0.03	0.01	0.19	0.09
CDA (RE)	1.00	1.00	1.00	1.00	1.00	1.00	1.00	1.00	1.00	1.00	1.00	1.00	1.00	1.00	1.00
CDA (PC)	0.93	0.95	0.94	0.94	0.96	0.93	0.95	0.94	0.94	0.96	0.93	0.95	0.94	0.94	0.96
ACA (PB)	0.17	0.48	0.35	5.33	0.64	0.03	0.35	0.31	2.41	0.23	0.13	0.57	0.12	4.98	0.18
ACA (SD)	0.78	0.05	0.01	0.24	0.11	0.77	0.06	0.01	0.23	0.11	0.78	0.05	0.01	0.24	0.11
ACA (SE)	0.75	0.05	0.01	0.23	0.11	0.76	0.06	0.01	0.23	0.11	0.75	0.05	0.01	0.23	0.11
ACA (RE)	**1.22**	**1.79**	**1.22**	**1.22**	**1.22**	**1.25**	**1.97**	**1.23**	**1.20**	**1.23**	**1.23**	**1.78**	**1.22**	**1.20**	**1.22**
ACA (PC)	0.93	0.95	0.95	0.94	0.95	0.94	0.94	0.95	0.94	0.96	0.93	0.96	0.96	0.94	0.95
FCS (PB)	0.21	0.43	0.50	5.11	0.65	0.08	0.68	0.09	2.35	0.73	0.17	0.56	0.34	4.64	0.09
FCS (SD)	0.77	0.05	0.01	0.24	0.11	0.83	0.06	0.01	0.25	0.11	0.78	0.05	0.01	0.24	0.11
FCS (SE)	0.77	0.05	0.01	0.23	0.11	0.83	0.07	0.01	0.25	0.12	0.77	0.05	0.01	0.23	0.11
FCS (RE)	1.25	1.89	1.24	1.24	1.24	1.35	2.37	1.35	1.30	1.34	1.25	1.90	1.24	1.23	1.24
FCS (PC)	0.94	0.94	0.95	0.94	0.96	0.95	0.96	0.95	0.95	0.96	0.94	0.96	0.95	0.93	0.95
CART (PB)	0.69	1.11	10.36	28.38	12.20	1.05	0.08	12.48	26.46	14.56	0.71	1.25	10.26	27.35	11.67
CART (SD)	0.71	0.05	0.01	0.18	0.10	0.71	0.06	0.01	0.19	0.10	0.72	0.05	0.01	0.19	0.10
CART (SE)	0.72	0.05	0.01	0.22	0.11	0.74	0.05	0.01	0.22	0.11	0.72	0.05	0.01	0.22	0.11
CART (RE)	1.18	1.65	1.18	1.16	1.18	1.21	1.85	1.21	1.15	1.20	1.18	1.65	1.18	1.15	1.17
CART (PC)	0.93	0.90	0.89	0.94	0.95	0.92	0.90	0.86	0.94	0.94	0.95	0.91	0.90	0.94	0.95
PAN (PB)	0.16	0.54	0.29	5.24	0.49	0.04	0.36	0.26	2.58	0.17	0.14	0.58	0.17	4.84	0.26
PAN (SD)	0.78	0.05	0.01	0.24	0.11	0.76	0.06	0.01	0.23	0.11	0.78	0.05	0.01	0.24	0.11
PAN (SE)	0.75	0.05	0.01	0.23	0.11	0.76	0.06	0.01	0.23	0.11	0.75	0.05	0.01	0.23	0.11
PAN (RE)	1.23	1.81	1.22	1.22	1.22	1.25	1.99	1.23	1.20	1.23	1.22	1.79	1.22	1.20	1.22
PAN (PC)	0.93	0.94	0.94	0.94	0.95	0.95	0.94	0.95	0.94	0.95	0.94	0.95	0.95	0.94	0.95

Missing proportion for each scenario is presented. True values are $\boldsymbol{\beta }= (31, -0.45, -0.07, 0.56, 0.4)$

Based on the simulation results, we find that all of the estimates from ACA, FCS, and PAN are unbiased. However, estimation bias emerges by using CART no matter the missingness of $\boldsymbol{Y}_i$ depends on observed outcomes, observed covariates, or both. Loss of efficiency exists in ACA, FCS, and PAN. There are negligible differences in the REs from ACA and PAN, whereas slightly more loss of efficiency is observed for FCS because of the variability introduced by MI methods. Therefore, we recommend ACA when missing data are observed in longitudinal outcomes only for LMMs, as it is computationally more efficient than PAN in general.

### Missing Data in Covariates Only

Although it is uncommon that longitudinal outcomes are complete, we intend to provide a comprehensive simulation analysis by assuming that missing values only exist in covariates in this section. Analogous to Sect. 5.2, we consider the missingness of $\boldsymbol{X}_i$ generated in three different ways.

At first, we assume that the missing values of $\boldsymbol{X}_{2,i}$ depend on other covariates $\boldsymbol{X}_{1,i}$, $\boldsymbol{X}_{3,i}$, and $\boldsymbol{X}_{4,i}$, all of which are fully observed. We propose a logistic model analogous to Eq. (1):

2 $$\begin{aligned} \boldsymbol{R}_{i} = \texttt { logit}(\gamma _{0,i} + \boldsymbol{\gamma }_{1,i} \boldsymbol{X}_{1,i} + \gamma _{3,i} \boldsymbol{X}_{3,i} + \gamma _{4,i} \boldsymbol{X}_{4,i}). \end{aligned}$$

Be aware that the $\gamma$’s in Eq. (2) are not necessarily identical to the counterparts in Eq. (1). The assignments of $\gamma$’s are based on the practical needs of missing proportion control. For the next case, we assume that the missing values of $\boldsymbol{X}_{2,i}$ only depend on $\boldsymbol{Y}_{i}$, inducing a similar missing data generation model:

$$\boldsymbol{R}_{i} = \texttt { logit}(\gamma _{0,i} + \boldsymbol{\gamma }_{5,i} \boldsymbol{Y}_{i}),$$

with an appropriate selection of $\boldsymbol{\gamma }_{5,i}$. Lastly, we model the missingness of $\boldsymbol{X}_{2,i}$ based on all observed values in the dataset; namely, $\boldsymbol{Y}_{i}$, $\boldsymbol{X}_{1,i}$, $\boldsymbol{X}_{3,i}$, and $\boldsymbol{X}_{4,i}$. The simulation results are shown in Table 3.

**Table 3:** Simulation results for missing values occurring in $\boldsymbol{X}_i$ only under the assumption of MAR, where the missingness of $\boldsymbol{X}_i$, respectively, depends on other fully observed covariates, observed responses, or both (from left to right)

	$\boldsymbol{R}\mid \boldsymbol{X}_{\text {obs}} \; (64\%)$					$\boldsymbol{R}\mid \boldsymbol{Y}_{{\text {obs}}} \; (57\%)$					$\boldsymbol{R}\mid \boldsymbol{Y}_{\text {obs}}, \boldsymbol{X}_{\text {obs}} \; (68\%)$				
	$\beta _0$	$\beta _1$	$\beta _2$	$\beta _3$	$\beta _4$	$\beta _0$	$\beta _1$	$\beta _2$	$\beta _3$	$\beta _4$	$\beta _0$	$\beta _1$	$\beta _2$	$\beta _3$	$\beta _4$
CDA (PB)	0.17	0.17	0.53	3.10	0.05	0.17	0.17	0.53	3.10	0.05	0.17	0.17	0.53	3.10	0.05
CDA (SD)	0.64	0.03	0.01	0.20	0.09	0.64	0.03	0.01	0.20	0.09	0.64	0.03	0.01	0.20	0.09
CDA (SE)	0.61	0.03	0.01	0.19	0.09	0.61	0.03	0.01	0.19	0.09	0.61	0.03	0.01	0.19	0.09
CDA (RE)	1.00	1.00	1.00	1.00	1.00	1.00	1.00	1.00	1.00	1.00	1.00	1.00	1.00	1.00	1.00
CDA (PC)	0.93	0.95	0.94	0.94	0.96	0.93	0.95	0.94	0.94	0.96	0.93	0.95	0.94	0.94	0.96
ACA (PB)	0.10	0.06	0.18	2.46	0.82	0.30	0.46	41.08	42.02	40.48	1.77	0.10	1.47	9.94	0.56
ACA (SD)	1.06	0.05	0.02	0.33	0.15	0.78	0.04	0.01	0.23	0.10	1.11	0.05	0.02	0.33	0.16
ACA (SE)	1.06	0.05	0.02	0.31	0.15	0.71	0.04	0.01	0.22	0.11	1.10	0.05	0.02	0.32	0.16
ACA (RE)	1.73	1.68	1.69	1.62	1.69	1.16	1.53	1.22	1.15	1.19	1.80	1.78	1.78	1.70	1.77
ACA (PC)	0.95	0.96	0.94	0.93	0.95	0.91	0.93	0.25	0.79	0.67	0.91	0.95	0.94	0.93	0.94
FCS (PB)	0.21	0.17	0.81	2.74	3.96	0.14	0.17	0.33	3.80	4.68	0.22	0.17	0.68	3.10	3.36
FCS (SD)	0.67	0.03	0.01	0.21	0.14	0.65	0.03	0.01	0.21	0.16	0.68	0.03	0.01	0.22	0.15
FCS (SE)	0.65	0.03	0.01	0.20	0.15	0.64	0.03	0.01	0.20	0.17	0.66	0.03	0.01	0.20	0.15
FCS (RE)	**1.06**	**1.00**	**1.06**	**1.05**	**1.61**	**1.04**	**1.00**	**1.05**	**1.04**	**1.87**	**1.07**	**1.00**	**1.07**	**1.06**	**1.70**
FCS (PC)	0.94	0.95	0.94	0.93	0.97	0.94	0.95	0.95	0.94	0.95	0.94	0.95	0.94	0.93	0.94
CART (PB)	0.13	0.17	0.12	4.89	4.33	0.04	0.17	1.43	5.68	18.02	0.12	0.17	0.33	5.24	5.43
CART (SD)	0.66	0.03	0.01	0.21	0.16	0.66	0.03	0.01	0.21	0.27	0.69	0.03	0.01	0.21	0.18
CART (SE)	0.62	0.03	0.01	0.19	0.11	0.62	0.03	0.01	0.19	0.10	0.62	0.03	0.01	0.19	0.11
CART (RE)	1.01	1.00	1.01	1.01	1.17	1.01	1.00	1.01	1.01	1.15	1.01	1.00	1.01	1.01	1.18
CART (PC)	0.94	0.95	0.93	0.93	0.82	0.93	0.95	0.93	0.93	0.54	0.92	0.95	0.93	0.92	0.75
PAN (PB)	0.20	0.17	0.73	3.26	64.08	0.19	0.17	1.08	2.89	74.42	0.17	0.17	0.67	3.06	68.28
PAN (SD)	0.65	0.03	0.01	0.21	0.06	0.66	0.03	0.01	0.21	0.05	0.66	0.03	0.01	0.21	0.05
PAN (SE)	0.63	0.03	0.01	0.19	0.12	0.63	0.03	0.01	0.19	0.12	0.63	0.03	0.01	0.19	0.12
PAN (RE)	1.03	1.00	1.03	1.03	1.34	1.03	1.00	1.03	1.03	1.34	1.04	1.00	1.04	1.03	1.36
PAN (PC)	0.93	0.95	0.94	0.94	0.37	0.93	0.95	0.94	0.94	0.11	0.92	0.95	0.94	0.94	0.28

Missing proportion for each scenario is presented. True values are $\boldsymbol{\beta }= (31, -0.45, -0.07, 0.56, 0.4)$

According to the simulation results, we do not recommend PAN as the estimate of $\beta _4$ is biased across all three scenarios. PAN fails since missing data emerge in covariates. Among ACA, FCS, and CART, we will carry out a case-by-case discussion. When the missingness of $\boldsymbol{X}_{2,i}$ depends only on observed responses, we do not observe bias in ACA, FCS, or CART. Pertaining to RE, we see obvious efficiency gain in FCS, whereas the efficiency gain from CART is the highest. However, we find PC for $\beta _4$ from CART is as low as $82\%$, bringing a great deal of concerns of the stability of the method. We hence recommend FCS for this scenario.

We next look into the scenario that the missingness of $\boldsymbol{X}_{2,i}$ depends on observed outcomes. We do not recommend ACA due to severe estimation bias. For the same reason, CART is not proper due to the bias in the estimate of $\beta _4$. Thus, we are in favor of FCS again for this scenario.

If the missingness of $\boldsymbol{X}_{2,i}$ depends on both observed responses and covariates, we find modest bias in the estimate of $\beta _3$ from ACA, so it does not seem to be a suitable method. The reduction of bias in ACA is primarily because the missingness of $\boldsymbol{X}_{2,i}$ is much more heavily dependent on fully observed covariates than observed outcomes in the missing data generation procedure. Accordingly, we almost do not see significant estimation bias in CART, either. Nonetheless, there is more efficiency gain in FCS than ACA, rendering that FCS is preferred. Though the efficiency gain in CART is the most of all, we do not recommend this method since the PC for $\beta _4$ is extensively low (i.e., $75\%$). However, CART may be a plausible alternative when the linearity of imputation model is questionable. To summarize, we give our recommendation to FCS when missing data occur in covariates only regardless of the pattern of missing data generation.

### Missing Data in Both Responses and Covariates

It is more common to have missing values existing in both longitudinal responses and covariates. In this section, we run the simulations under this general assumption, and compare the performances of ACA, FCS, CART, and PAN under a variety of missing data generation schemes.

At first, we consider that the missingness of $\boldsymbol{X}_{2,i}$ depends only on other fully observed covariates (i.e., Eq. (2)), while the missing values of $\boldsymbol{Y}_{i}$ are generated in the following two different ways:

1. The missing values in $\boldsymbol{Y}_i$ are based on all observed covariates;
2. The missing values in $\boldsymbol{Y}_i$ are based on not only observed covariates but also on previously observed responses.

Since the missingness of $\boldsymbol{X}_{2,i}$ depends on the data with no missing value, we, in practice, always generate those missing data first, followed by generating missing values in longitudinal responses. The simulation results are given in Table 4.

**Table 4:** Simulation results for missing values occurring in $\boldsymbol{Y}_i$ and $\boldsymbol{X}_{2,i}$, where the missingness of $\boldsymbol{X}_{2,i}$ depends only on fully observed covariates, and the missingness of $\boldsymbol{Y}_i$, respectively, depends on other fully observed covariates or on both of observed covariates and responses (from left to right)

	$\boldsymbol{R}\mid \boldsymbol{X}_{\text {obs}}; \boldsymbol{S}\mid \boldsymbol{X}_{\text {obs}} \; (67\%)$					$\boldsymbol{R}\mid \boldsymbol{X}_{\text {obs}}; \boldsymbol{S}\mid \boldsymbol{X}_{\text {obs}}, \boldsymbol{Y}_{\text {obs}} \; (65\%)$				
	$\beta _0$	$\beta _1$	$\beta _2$	$\beta _3$	$\beta _4$	$\beta _0$	$\beta _1$	$\beta _2$	$\beta _3$	$\beta _4$
CDA (PB)	0.17	0.17	0.53	3.10	0.05	0.17	0.17	0.53	3.10	0.05
CDA (SD)	0.64	0.03	0.01	0.20	0.09	0.64	0.03	0.01	0.20	0.09
CDA (SE)	0.61	0.03	0.01	0.19	0.09	0.61	0.03	0.01	0.19	0.09
CDA (RE)	1.00	1.00	1.00	1.00	1.00	1.00	1.00	1.00	1.00	1.00
CDA (PC)	0.93	0.95	0.94	0.94	0.96	0.93	0.95	0.94	0.94	0.96
ACA (PB)	0.20	0.46	0.22	5.55	0.51	0.06	0.34	0.67	4.89	0.51
ACA (SD)	0.90	0.05	0.01	0.28	0.13	0.88	0.05	0.01	0.27	0.14
ACA (SE)	0.91	0.06	0.01	0.26	0.13	0.92	0.05	0.01	0.27	0.14
ACA (RE)	1.48	1.95	1.45	1.39	1.44	1.49	1.87	1.47	1.41	1.58
ACA (PC)	0.96	0.96	0.94	0.93	0.96	0.95	0.96	0.96	0.95	0.96
FCS (PB)	0.23	0.01	0.84	3.72	1.27	0.19	0.22	0.45	4.03	3.20
FCS (SD)	0.72	0.05	0.01	0.22	0.13	0.72	0.05	0.01	0.21	0.13
FCS (SE)	0.72	0.05	0.01	0.22	0.13	0.71	0.05	0.01	0.21	0.14
FCS (RE)	**1.17**	**1.69**	**1.17**	**1.15**	**1.45**	**1.16**	**1.68**	**1.16**	**1.14**	**1.56**
FCS (PC)	0.95	0.95	0.96	0.94	0.96	0.95	0.96	0.95	0.95	0.96
CART (PB)	0.45	0.16	6.53	17.08	8.74	0.44	0.24	6.69	17.22	14.23
CART (SD)	0.68	0.05	0.01	0.19	0.12	0.68	0.04	0.01	0.19	0.12
CART (SE)	0.68	0.04	0.01	0.20	0.12	0.68	0.04	0.01	0.20	0.13
CART (RE)	1.12	1.45	1.12	1.08	1.35	1.12	1.44	1.12	1.09	1.47
CART (PC)	0.95	0.92	0.92	0.93	0.95	0.95	0.94	0.92	0.95	0.94
PAN (PB)	0.20	0.13	0.61	3.66	44.68	0.22	0.16	0.17	4.29	48.80
PAN (SD)	0.71	0.04	0.01	0.22	0.07	0.70	0.04	0.01	0.22	0.07
PAN (SE)	0.70	0.04	0.01	0.21	0.12	0.69	0.04	0.01	0.21	0.12
PAN (RE)	1.14	1.53	1.14	1.12	1.28	1.14	1.44	1.13	1.12	1.33
PAN (PC)	0.94	0.95	0.95	0.93	0.74	0.95	0.95	0.94	0.95	0.70

Missing proportion for each scenario is presented. True values are $\boldsymbol{\beta }= (31, -0.45, -0.07, 0.56, 0.4)$

The simulation results imply that the estimates from both ACA and FCS are unbiased, whereas the efficiency gains from FCS are consistently higher than the counterparts from ACA. Neither CART nor PAN appears to be an appropriate option owing to estimation bias. Thus, we conclude that FCS outperforms ACA for these two scenarios.

Next, we assume that the missingness of $\boldsymbol{X}_{i,2}$ depends not only on other fully observed covariates, but on observed responses as well. We again consider two different missing data mechanisms for $\boldsymbol{Y}_{i}$ identical to the preceding scenario. We present the simulation results in Table 5.

**Table 5:** Simulation results for missing values occurring in $\boldsymbol{Y}_i$ and $\boldsymbol{X}_{2,i}$, where the missingness of $\boldsymbol{X}_{2,i}$ depends on both of observed covariates and responses, and the missingness of $\boldsymbol{Y}_i$ depends on fully observed covariates or on both of observed covariates and responses (from left to right)

	$\boldsymbol{R}\mid \boldsymbol{X}_{\text {obs}}, \boldsymbol{Y}_{\text {obs}}; \boldsymbol{S}\mid \boldsymbol{X}_{\text {obs}} \; (67\%)$					$\boldsymbol{R}\mid \boldsymbol{X}_{\text {obs}}, \boldsymbol{Y}_{\text {obs}}; \boldsymbol{S}\mid \boldsymbol{X}_{\text {obs}}, \boldsymbol{Y}_{\text {obs}} \; (67\%)$				
	$\beta _0$	$\beta _1$	$\beta _2$	$\beta _3$	$\beta _4$	$\beta _0$	$\beta _1$	$\beta _2$	$\beta _3$	$\beta _4$
CDA (PB)	0.17	0.17	0.53	3.10	0.05	0.17	0.17	0.53	3.10	0.05
CDA (SD)	0.64	0.03	0.01	0.20	0.09	0.64	0.03	0.01	0.20	0.09
CDA (SE)	0.61	0.03	0.01	0.19	0.09	0.61	0.03	0.01	0.19	0.09
CDA (RE)	1.00	1.00	1.00	1.00	1.00	1.00	1.00	1.00	1.00	1.00
CDA (PC)	0.93	0.95	0.94	0.94	0.96	0.93	0.95	0.94	0.94	0.96
ACA (PB)	1.35	18.88	0.74	5.68	3.34	1.67	30.17	6.84	10.14	15.25
ACA (SD)	0.93	0.06	0.01	0.29	0.13	1.01	0.06	0.02	0.30	0.15
ACA (SE)	0.93	0.06	0.01	0.28	0.14	0.97	0.06	0.01	0.30	0.15
ACA (RE)	1.51	2.01	1.51	1.49	1.51	1.59	2.26	1.61	1.58	1.69
ACA (PC)	0.92	0.67	0.94	0.95	0.97	0.90	0.44	0.93	0.94	0.94
FCS (PB)	**0.19**	**0.33**	**0.64**	**3.32**	**5.12**	**0.13**	**0.95**	**0.03**	**4.73**	**4.65**
FCS (SD)	0.69	0.04	0.01	0.22	0.13	0.75	0.05	0.01	0.23	0.15
FCS (SE)	0.68	0.04	0.01	0.21	0.14	0.76	0.05	0.01	0.23	0.16
FCS (RE)	1.12	1.51	1.11	1.11	1.52	1.23	1.81	1.23	1.22	1.78
FCS (PC)	0.94	0.96	0.95	0.94	0.96	0.96	0.93	0.94	0.95	0.97
CART (PB)	0.26	0.28	4.24	12.44	8.78	0.67	1.32	9.92	24.88	15.04
CART (SD)	0.67	0.04	0.01	0.19	0.13	0.69	0.05	0.01	0.19	0.14
CART (SE)	0.66	0.04	0.01	0.20	0.13	0.71	0.04	0.01	0.21	0.15
CART (RE)	1.08	1.36	1.08	1.06	1.42	1.16	1.58	1.17	1.13	1.70
CART (PC)	0.94	0.93	0.94	0.95	0.94	0.95	0.91	0.90	0.92	0.95
PAN (PB)	0.19	0.39	0.81	3.31	55.09	0.15	2.04	0.26	4.80	62.09
PAN (SD)	0.68	0.04	0.01	0.21	0.06	0.75	0.04	0.01	0.23	0.06
PAN (SE)	0.67	0.04	0.01	0.20	0.12	0.73	0.05	0.01	0.22	0.12
PAN (RE)	1.09	1.42	1.09	1.08	1.30	1.19	1.61	1.19	1.18	1.36
PAN (PC)	0.93	0.96	0.95	0.95	0.56	0.95	0.95	0.95	0.95	0.46

Missing proportion for each scenario is presented. True values are $\boldsymbol{\beta }= (31, -0.45, -0.07, 0.56, 0.4)$

According the simulation results, except for FCS, all of the remaining methods, ACA, CART, or PAN, produce biased estimates. Therefore, our recommendation is given to FCS. The failure of ACA is primarily because the missing data occurring in covariates depend on the observed longitudinal responses.

To conclude, under the assumption of MAR, PAN outperforms FCS when missing values are observed in longitudinal outcomes only. However, it does not present obvious advantage against ACA. When there are missing values only in covariates, we prefer FCS since it leads to higher relative efficiency than ACA. However, the estimates by using PAN are biased under this scenario. When both longitudinal outcomes and covariates have missing values, the estimates from both ACA and FCS are unbiased when the missing values in covariates are independent of longitudinal outcomes. However, FCS outperforms ACA in terms of efficiency gain. CART has the highest efficiency gain based on the simulation results, but the low probability coverage for some estimates may cause the lack of estimation reliability. Hence, our preference is given to FCS. While the missing values in covariates depend on longitudinal outcomes, only the estimates from FCS are unbiased, rendering it a preferred method among all of the considered methods.

At the end of this section, we would like to address that missing proportion is not our major concern in the present study, however, it may be related to the performance of missing data methods. The missing data pattern for PPMI study (which will be introduced explicitly in Sect. 6) is closest to the first scenario from Table 4. We hence take it as an example, and present the results in Table 8 (shown in Appendix 1), where we find that our recommendation remains the same.

## Real Data Analysis

In this section, we apply the missing data methods that are considered in this paper to a longitudinal dataset of PD. Specifically, We use data from the Parkinson’s Progression Markers Initiative (PPMI) which is a longitudinal study of biomarkers for early PD patients [51]. Data were downloaded in January 2018. Existing literature suggests that change of frontal lobe volume may be related to cognitive impairment in PD [52]. The study aims, diagnostic criteria, methods, and subject retention were previously described by Marek et al [53]. Our research goal is to explore the association between frontal lobe volume (frontal ROI, where ROI is the abbreviation of region of interest) obtained by magnetic resonance imaging (MRI) and cognitive decline of PD. In this study, we consider baseline frontal ROI as the primary predictor. To account for differences in brain size, baseline frontal ROI is divided by the intracranial volume prior to model fit.

According to the collection of PPMI data, missing data exist in frontal ROI. The main reason of missing frontal ROI is that some participating sites (of PPMI) do not have facilities to perform research quality MRI. Therefore, the missingness of frontal ROI is mainly due to study design, and thus the assumption of MCAR is plausible for frontal ROI. Besides, we have missing data in the longitudinal outcomes. The missing data in the longitudinal outcomes are assumed to be MAR, which will be demonstrated explicitly below. In addition to the initial visit, each participant in the cohort has at most five follow-up visits. Since different participants may join the study at different time points, some individuals may have missed a few longitudinal outcomes not due to dropouts or any personal reasons, but simply because the follow-up visit is not yet due. Thus, we shrink the study period up to two follow-up visits. We fit the final PPMI data using an LMM, where no missing data exist in other three covariates; namely, time (temporal), baseline age (fixed), and gender (fixed). The baseline demographics of the PPMI data, together with the missing proportion of each variable, are given in Table 6.

**Table 6:** Baseline demographics for the PPMI data; We report mean ± SD for continuous variables

Variable	Sample size (423)	
	Statistic	Missing (%)
Age	$61.66 \pm 9.71$	0.00
Gender	34.52% (female)	0.00
Frontal ROI	$0.11 \pm 0.01$	62.17
MoCA	$27.13 \pm 2.32$	0.71

**Fig. 1:**
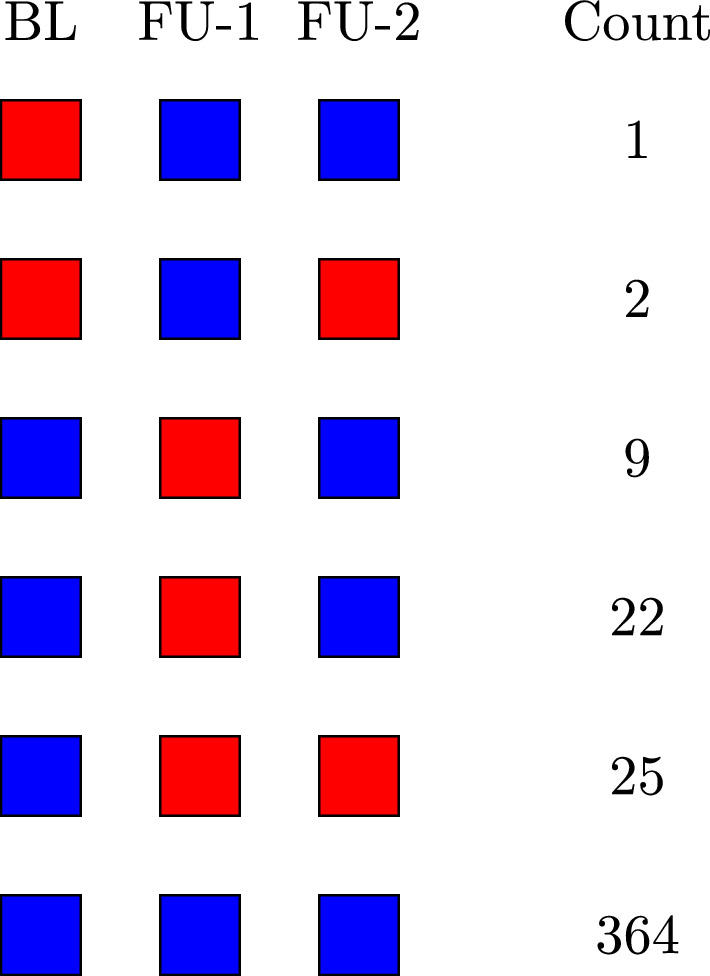
An illustration of missing patterns of MoCA with BL, FU-1, and FU-2, respectively, representing baseline, the first follow-up, and the second follow-up. Blue and red squares indicate observed (blue) and missing (red) data, respectively

The longitudinal outcome in our study is Montreal Cognitive Assessment (MoCA) score, which is a global cognitive ability measure and collected at each visit. The MoCA score ranges from 0 to 30, with a higher score indicating better cognitive function. The PPMI cohort includes only newly diagnosed participants with mean disease duration of 6.7 months [53]. Our previous findings show that $91.5 \%$ of the PPMI participants are cognitively normal by clinicians’ diagnosis at baseline [54]. It is unlikely that participants are missing MoCA due to unobserved poor MoCA scores in the first two years. Thus, there are no scientific reasons against the MAR assumption for the MoCA outcome. Specifically, the missing proportions of MoCA at the baseline and two followups are $0.71 \%$, $7.33 \%$, and $11.58 \%$, respectively, where the pattern of missingness is not monotone. A graphic illustration is presented in Fig. 1.

We consider an LMM with time, baseline age, gender, and frontal ROI as regressors. By implementing ACA, FCS, CART, and PAN, we summarize the estimation results in Table 7. We find similar estimates for the intercept, time and age across all four methods. The estimate for gender by using PAN is notably greater than those estimated by the other three methods, while the estimate of gender from FCS is the smallest. It is obvious that the estimate of frontal ROI from PAN is biased, which is consistent with our simulation summary that PAN does not perform well when missing values exist in covariates. On the other hand, the estimates for frontal ROI from FCS and CART are almost identical, slightly greater than that from ACA. In general, we do not see significant difference in the estimation results among ACA, FCS, and CART.

**Table 7:** Analysis results of LMM taking time, baseline age, gender, and frontal ROI as covariates

Parameter	Name	Missing data methods			
		ACA	FCS	CART	PAN
Intercept	EST	30.917	30.549	30.534	30.951
	SE	1.138	0.863	0.826	0.824
Time	EST	$-0.454$	$-0.424$	$-0.409$	$-0.429$
	SE	0.103	0.066	0.065	0.065
Age	EST	$-0.069$	$-0.068$	$-0.069$	$-0.078$
	SE	0.017	0.014	0.012	0.023
Gender	EST	0.563	0.489	0.545	0.653
	SE	0.329	0.247	0.233	0.238
Frontal ROI	EST	0.363	0.441	0.440	0.132
	SE	0.170	0.202	0.132	0.151

Based on our analysis results, we see that PAN is not a recommended method though the analysis model of the real data has multi-levels. The occurrence of missing data in frontal ROI causes estimation bias by using PAN. On the other hand, ACA, FCS, and CART provide close estimates for the regression coefficients. When frontal ROI is MCAR and MoCA is MAR, our simulations suggest that the estimates from ACA are unbiased. Thus, FCS is supposed to provide unbiased estimation provided that the assumption of LMM is valid. Additionally, the SEs of the MI methods (FCS and CART) are smaller than the counterparts of ACA in general. However, it is challenging to assess which of the two single-level MI methods provides a better fit to the data because of the lack of golden standard. Interestingly, our results imply that CART is a competitive approach in real data analysis. There are two possible reasons:

1. In practice, neither the true analysis model nor the true imputation model is known. There may exist non-linear relationships between the outcome variable and the covariate variables, which are difficult to be captured by LMM or FCS. Nonparametric methods, like CART, are more robust for non-linear models;
2. The assumption of MAR itself is difficult to check. If the missing data are MNAR, the performances among different kinds of MI methods have not yet been explored. The assumptions of the missing data in the present study are based on professional expertise, but not justified through rigorous statistical tests.

Nevertheless, real data application suggests that CART can be always served as an alternative in practical analyses, as this approach is model-free, and hence may be more robust than parametric methods in complex settings.

## Conclusion and Discussion

Finally, we address some concluding remarks and carry out some discussions about the paper. Based on extensive simulations, we observe that CART is not the most attractive option when the analysis model is known to be LMM. PAN is a reliable approach and it outperforms FCS when there is no missing value in covariates. However, it does not lead to efficiency gain relative to ACA. Under the assumption of MCAR, we prefer ACA when missing data exist in responses only, but recommend FCS when missing data exist in covariates or in both covariates and responses. Under the assumption of MAR, when missing data only exist in longitudinal outcomes, we do not observe bias in either ACA, FCS, or PAN, but ACA and PAN present higher efficiency gains than FCS. For missing data only exist in covariates where the missingness depends on longitudinal outcomes, we find severe bias in some estimates from ACA, so recommend FCS, all the estimates from which are unbiased. For the most general setting that missing data are observed in both longitudinal outcomes and covariates, we consider various scenarios but arrive at a unified conclusion: FCS. If the missing data in covariates do not depend on longitudinal outcomes, FCS outperforms ACA reflected in more efficiency gain; otherwise (i.e., missing data of covariates depend on both of longitudinal outcomes and covariates), FCS is preferred as estimation bias is found in ACA. It is worthy of noting that for the last two scenarios, our recommendations do not change against the missing data generation processes of longitudinal outcomes (i.e., depending only on observed covariates or on both observed covariates and outcomes) as long as the assumption of MAR holds.

Recent developments in DL algorithms for imputation have significantly advanced the ability to handle complex missing data patterns. Techniques such as generative adversarial networks [GANs, 20] and variational autoencoders [VAEs, 55] that leverage non-linear relationships and high-capacity function approximators have been proposed to model missing data distributions. Although DL-based methods are seemingly powerful for large and high-dimensional datasets, they require large datasets to generalize well-trained models, making them less effective for small- or moderate-sample data. Furthermore, the performance of DL-based MI approaches can be highly sensitive to hyperparameter tuning, architecture selection, and missing data mechanisms, which requires a systematic study for precise evaluation. Since this paper mainly focuses on the comparison between ACA and traditional MI methods, we carry out a preliminary study of imputation based on multi-layer perceptron [MLP, 56] for the scenario where missing data (with varying missing proportions) exist in both longitudinal outcomes and covariates, where the missingness depends on observed covariates only (Appendix 1). The results are added to Table 8.

It is evident that model-based MI methods may lead to inaccurate inference when imputation models are incorrectly posed [9]. In the present study, the analysis model is LMM under the assumption of normality, and the imputation model of single-level and multilevel MI methods are, respectively, based on linear regression and LMM. The imputation models are seemingly correct for both classes of methods, hence the MI methods, especially FCS, lead to accurate estimates. However, if the imputation model is not correctly specified, inference may become invalid; see an example in Appendix 2. Hence, the validity of parametric MI methods heavily relies on the correct specification of the imputation model throughout the analysis.

One of the limitations of the present work is that the comparison between ACA and MI methods is mainly done assuming linear relationships in our models. Given the growing evidence that cognitive endpoints in PD change non-linearly over time, one of our planned future work is to assess the performance of various MI methods for non-linear mixed-effects models (NLMEMs). Many of the conclusions from this study may still apply, but rigorous, systematic investigations that fully and deeply examine each missing data scenario are needed to provide the most accurate recommendations. Moreover, since existing literature suggests that DL-based approaches can capture non-linear relationships between variables, we run an example experiment on MLP, where a quadratic term is considered. The experiment results are presented in Appendix 3, but a comprehensive study will be conducted in our future works.

In addition, the LMM-based study in the present work may limit the generalizability of our findings to generalized linear mixed-effects models (GLMMs). The performance of MI in the context of GLMMs is influenced by several factors, including the proportion and mechanism of missing data, as well as the complexity of the structure of random effects. While MI has the potential to produce unbiased and efficient parameter estimates when the imputation model is correctly specified, its application to GLMMs presents additional challenges due to the non-linear link functions and distributional assumptions inherent in these models. Standard MI techniques may struggle to fully capture the dependency structures introduced by random effects, potentially leading to biased inference. Therefore, a rigorous and systematic investigation is necessary to assess the effectiveness of MI methods for GLMMs. Future work is needed for conducting comprehensive simulation studies and empirical evaluations in order to provide a more thorough understanding of MI performance in this setting.

While MI methods remain operational if the missing data are MNAR, they perform better under the MAR assumption [25]. Therefore, MAR is assumed throughout this study, which is another limitation of this paper because MNAR is common in practice. A recent study [57] suggested that specific model adjustment and sensitivity analyses would help optimize the performance of MI methods in MNAR situations. Besides, with the rapid development of DL algorithms in the recent yeas, DL-based imputation techniques have become promising alternatives to handling missing data that are MNAR, such as the deep generative imputation models developed from variational autoencoders [58, 59] and the causally aware imputation via learning missing data mechanisms [60]. However, the performance of DL algorithms for MI may vary significantly depending on specific datasets, and the inconsistency in performance may be attributed to various factors, which deserves further investigation in our future work. Structural missingness presents a significant challenge; however, it is not addressed in this study due to its non-random nature. We conceptualize structural missingness in two ways. First, it can be regarded as a type of MNAR, which is discussed earlier. Second, structural missingness may result from logical considerations. In such cases, the application of MI requires careful scrutiny to ensure that the imputed values align with the underlying rationale. To the best of our knowledge, research on this issue remains limited, underscoring the need for further in-depth investigation.

## Appendix A: Simulation Results for Various Missing Proportions

In addition to the traditional MI methods considered in the paper, we apply an MLP-based method to conduct a preliminary investigation of DL algorithms for missing data imputation. MLP is a type of neural network that consists of multiple layers of interconnected neurons: an input layer, a hidden layer, and an output layer. The rectified linear unit (ReLU) activation is used for the input layer, whereas the linear activation is used for the output layer. The adaptive moment estimation (Adam) algorithm is employed for optimization. The implementation is done through keras3 [61] and tensorflow [62] packages that interface R and Python. It is worth noting that MLP creates completely new data even for those that are not missing. We only take the imputed values for the missing data, but keep the observed values from the original dataset to create the imputed dataset for fitting LMM.

From Table 8, we do not observe estimation bias in ACA or FCS. For each method, more efficiency loss takes place with the increase of missing proportion. Nonetheless, the efficiency gain in FCS is consistently higher than that in ACA given fixed missing data proportion. Thus, FCS remains preferred relative to ACA for this missing data scenario. However, it is worthy of mentioning that the estimates from CART are only notably biased when the missing proportion is modest or high. For small missing proportion, the bias from CART is not too obvious, rendering it a potential alternative in the study.

Although the results in Table 8 suggest that MLP produces biased estimates for all three scenarios, we do not want to draw any conclusions about the performance of DL-based imputation methods. Instead, a systematic study including extensive sensitivity analyses under various settings is needed to make an accurate assessment.

**Table 8:** Simulation results for missing values occurring in $\boldsymbol{Y}_i$ and $\boldsymbol{X}_{2,i}$, where the missingness of $\boldsymbol{X}_{2,i}$ depends only on fully observed covariates, and the missingness of $\boldsymbol{Y}_i$ depends on other fully observed covariates, too

	$\boldsymbol{R}\mid \boldsymbol{X}_{{\text {obs}}}; \boldsymbol{S}\mid \boldsymbol{X}_{{\text {obs}}} \; (35\%)$					$\boldsymbol{R}\mid \boldsymbol{X}_{{\text {obs}}}; \boldsymbol{S}\mid \boldsymbol{X}_{{\text {obs}}} \; (46\%)$					$\boldsymbol{R}\mid \boldsymbol{X}_{{\text {obs}}}; \boldsymbol{S}\mid \boldsymbol{X}_{{\text {obs}}} \; (67\%)$				
	$\beta _0$	$\beta _1$	$\beta _2$	$\beta _3$	$\beta _4$	$\beta _0$	$\beta _1$	$\beta _2$	$\beta _3$	$\beta _4$	$\beta _0$	$\beta _1$	$\beta _2$	$\beta _3$	$\beta _4$
CDA (PB)	0.17	0.17	0.53	3.10	0.05	0.17	0.17	0.53	3.10	0.05	0.17	0.17	0.53	3.10	0.05
CDA (SD)	0.64	0.03	0.01	0.20	0.09	0.64	0.03	0.01	0.20	0.09	0.64	0.03	0.01	0.20	0.09
CDA (SE)	0.61	0.03	0.01	0.19	0.09	0.61	0.03	0.01	0.19	0.09	0.61	0.03	0.01	0.19	0.09
CDA (RE)	1.00	1.00	1.00	1.00	1.00	1.00	1.00	1.00	1.00	1.00	1.00	1.00	1.00	1.00	1.00
CDA (PC)	0.93	0.95	0.94	0.94	0.96	0.93	0.95	0.94	0.94	0.96	0.93	0.95	0.94	0.94	0.96
ACA (PB)	0.28	0.01	0.86	5.24	1.16	0.25	0.13	0.60	5.72	1.15	0.20	0.46	0.22	5.55	0.51
ACA (SD)	0.72	0.04	0.01	0.22	0.10	0.78	0.04	0.01	0.23	0.11	0.90	0.05	0.01	0.28	0.13
ACA (SE)	0.71	0.04	0.01	0.21	0.10	0.75	0.04	0.01	0.22	0.11	0.91	0.06	0.01	0.26	0.13
ACA (RE)	1.15	1.31	1.14	1.12	1.14	1.22	1.45	1.21	1.18	1.20	1.48	1.95	1.45	1.39	1.44
ACA (PC)	0.94	0.95	0.94	0.93	0.96	0.94	0.95	0.94	0.93	0.96	0.96	0.96	0.94	0.93	0.96
FCS (PB)	0.18	0.04	0.61	3.14	0.44	0.17	0.44	0.55	3.19	0.23	0.23	0.01	0.84	3.72	1.27
FCS (SD)	0.66	0.03	0.01	0.21	0.10	0.70	0.04	0.01	0.21	0.11	0.72	0.05	0.01	0.22	0.13
FCS (SE)	0.65	0.04	0.01	0.20	0.10	0.66	0.04	0.01	0.20	0.11	0.72	0.05	0.01	0.22	0.13
FCS (RE)	1.05	1.24	1.05	1.04	1.14	1.08	1.33	1.08	1.06	1.20	1.17	1.69	1.17	1.15	1.45
FCS (PC)	0.93	0.96	0.95	0.93	0.96	0.93	0.94	0.94	0.94	0.96	0.95	0.95	0.96	0.94	0.96
CART (PB)	0.08	0.07	2.15	8.00	2.35	0.17	0.40	3.20	10.62	3.40	0.45	0.16	6.53	17.08	8.74
CART (SD)	0.65	0.04	0.01	0.20	0.10	0.68	0.04	0.01	0.20	0.10	0.68	0.05	0.01	0.19	0.12
CART (SE)	0.64	0.03	0.01	0.19	0.10	0.65	0.04	0.01	0.20	0.11	0.68	0.04	0.01	0.20	0.12
CART (RE)	1.04	1.18	1.04	1.03	1.12	1.05	1.24	1.05	1.04	1.17	1.12	1.45	1.12	1.08	1.35
CART (PC)	0.94	0.95	0.94	0.94	0.95	0.93	0.93	0.93	0.94	0.95	0.95	0.92	0.92	0.93	0.95
PAN (PB)	0.15	0.13	0.45	2.87	17.20	0.17	0.33	0.52	3.39	24.50	0.20	0.13	0.61	3.66	44.68
PAN (SD)	0.67	0.03	0.01	0.21	0.08	0.69	0.04	0.01	0.21	0.08	0.71	0.04	0.01	0.22	0.07
PAN (SE)	0.64	0.03	0.01	0.20	0.10	0.66	0.04	0.01	0.20	0.11	0.70	0.04	0.01	0.21	0.12
PAN (RE)	1.05	1.20	1.05	1.04	1.13	1.07	1.27	1.07	1.06	1.17	1.14	1.53	1.14	1.12	1.28
PAN (PC)	0.93	0.95	0.95	0.94	0.95	0.93	0.95	0.94	0.94	0.91	0.94	0.95	0.95	0.93	0.74
MLP (PB)	1.81	1.57	15.09	10.54	26.04	2.39	1.48	19.90	14.48	18.82	4.26	3.00	34.71	22.82	4.13
MLP (SD)	0.61	0.04	0.01	0.19	0.26	0.61	0.05	0.01	0.19	0.27	0.50	0.07	0.01	0.16	0.37
MLP (SE)	0.56	0.03	0.01	0.17	0.15	0.54	0.02	0.01	0.17	0.14	0.48	0.02	0.01	0.15	0.15
MLP (RE)	0.92	0.90	0.92	0.92	1.62	0.88	0.87	0.88	0.88	1.60	0.79	0.79	0.79	0.79	1.66
MLP (PC)	0.80	0.77	0.74	0.90	0.72	0.70	0.69	0.59	0.89	0.70	0.24	0.47	0.06	0.85	0.56

Different missing proportions are considered. True values are $\boldsymbol{\beta }= (31, -0.45, -0.07, 0.56, 0.4)$

## Appendix B: Example of Invalid Inference by Using MI

Suppose that the analysis model is an LMM given by

$$Y_{ij} = \beta _0 + b_{i} + \beta _1 X_{1,ij} + \beta _2 X_{2,i} + \beta _3 X_{3,i} + \beta _4 X_{4,i} + \varepsilon _{ij}.$$

Assume that $X_{2,i}$ is a fixed covariate dependent on an auxiliary variables $X_{5,i}$ and $X_{6,i}$ in the form of $X_{2,i} = X_{5,i}^{8/3} + \exp \{X_{6,i} + 1\}$, where $X_{5,i} \sim \mathcal {N}(61,10)$ and $X_{6,i} \sim {{\text {Ber}}}(0.35)$. Besides, $X_{3,i}$ is non-linear in $X_{5,i}$ and $X_{6,i}$ as $X_{3,i} \sim 1/\sqrt{X_{5,i}} + 5 \times X_{i,6}$. Although $X_{5,i}$ and $X_{6,i}$ do not appear in the analysis model, in practice, researchers are suggested to include all possible covariates (i.e., available information) in the imputation procedure, rendering that the imputation model is seriously misspecified.

We conduct a simulation analysis analogous to that in Sect. 5.4. We use the same true values for $\boldsymbol{\beta }$, where the other settings are kept the same, too. The missing data in longitudinal outcomes and covariates are generated based on fully observed covariates. The simulation results via CDA, ACA, and FCS are presented in Table 9, where we find invalid inferences from all MI methods.

**Table 9:** Simulation results for misspecified imputation model (non-linear relationships)

	$\beta _0$	$\beta _1$	$\beta _2$	$\beta _3$	$\beta _4$
CDA (PB)	0.11	0.17	0.00	0.62	0.03
CDA (SD)	0.37	0.03	0.00	0.04	0.09
CDA (SE)	0.35	0.03	0.00	0.04	0.09
CDA (RE)	1.00	1.00	1.00	1.00	1.00
CDA (PC)	0.93	0.95	0.94	0.94	0.96
ACA (PB)	0.17	0.06	0.00	0.51	0.51
ACA (SD)	0.66	0.07	0.00	0.07	0.17
ACA (SE)	0.64	0.07	0.00	0.07	0.17
ACA (RE)	1.84	2.53	1.97	1.79	1.90
ACA (PC)	0.93	0.95	0.95	0.94	0.94
FCS (PB)	7637.55	15019.94	57.83	100.39	2953.34
FCS (SD)	121.36	32.53	0.00	7.83	14.58
FCS (SE)	199.80	40.51	0.00	19.40	49.48
FCS (RE)	570.83	1424.44	524.88	515.25	549.17
FCS (PC)	0.00	0.64	0.00	1.00	1.00
CART (PB)	7641.07	14990.00	57.83	86.60	2976.47
CART (SD)	121.73	32.14	0.00	7.88	18.79
CART (SE)	188.75	32.45	0.00	19.33	48.70
CART (RE)	539.26	1140.84	516.99	513.42	540.56
CART (PC)	0.00	0.45	0.00	1.00	1.00
PAN (PB)	7327.24	6358.86	57.54	79.63	3341.81
PAN (SD)	104.74	14.34	0.00	7.83	14.94
PAN (SE)	191.65	32.27	0.00	19.41	49.26
PAN (RE)	547.54	1134.80	522.24	515.60	546.75
PAN (PC)	0.00	1.00	0.00	1.00	1.00

True values are $\boldsymbol{\beta }= (31, -0.45, -0.07, 0.56, 0.4)$

## Appendix C: Example of NLMEM Application by Using DL

Suppose that the analysis model is a NLMEM given by

$$Y_{ij} = \beta _0 + b_{i} + \beta _1 X_{1,ij} + \beta _2 X_{2,i} + \beta _3 X_{3,i} + \beta _4 X^2_{4,i} + \varepsilon _{ij},$$

where a quadratic function is applied to the predictor $X_{4,i}$. The missing data generation is identical to the first case of Sect. 5.4, as it is closest to the missing data scenario of the PPMI study, i.e., missing data exist in both longitudinal outcomes and covariates under the MAR assumption. This setting is the same as that for the sensitivity analyses in Appendix 1 as well.

To clarify, this experiment is different from that in Appendix 2 which considers a non-linear structure in the imputation model and aims to show that misspecified imputation model leads to invalid inference. The experiment in this section focuses on the non-linear relationships in the analysis model. For brevity, we report the results for CDA and MLP only in Table 10. When conducting CDA, we pretend that we know the correct covariate to use; That is, we use $X^2_{4,i}$ to fit the model. However, when testing MLP, we pretend that we do not know the correct analysis model, so $X_{4,i}$ is used in the training. The results indicate that MLP perform well to estimate $\beta _0$ and $\beta _3$, but the estimate of $\beta _4$ is severely biased. One possible reason related to the bias is that the dataset may not be large enough to train the deep learning model well. Nevertheless, this example experimental result is far from being enough to evaluate the performance of DL-based methods for NLMEM. A systematic studies with extensive sensitivity analysis is needed in future work.

**Table 10:** Simulation results for missing values occurring in $\boldsymbol{Y}_i$ and $\boldsymbol{X}_{2,i}$, where the missingness of $\boldsymbol{X}_{2,i}$ depends only on fully observed covariates, and the missingness of $\boldsymbol{Y}_i$ depends on other fully observed covariates, too

	$\beta _0$	$\beta _1$	$\beta _2$	$\beta _3$	$\beta _4$
CDA (PB)	0.10	0.02	0.31	1.40	1.03
CDA (SD)	0.59	0.03	0.01	0.18	0.07
CDA (SE)	0.62	0.03	0.01	0.19	0.06
CDA (RE)	1.00	1.00	1.00	1.00	1.00
CDA (PC)	0.97	0.94	0.95	0.95	0.95
MLP (PB)	1.16	2.00	19.48	10.64	100.03
MLP (SD)	0.54	0.05	0.01	0.18	0.11
MLP (SE)	0.56	0.02	0.01	0.17	0.09
MLP (RE)	0.91	0.87	0.92	0.92	1.44
MLP (PC)	0.90	0.68	0.64	0.94	0.02

Different missing proportions are considered. True values are $\boldsymbol{\beta }= (31, -0.45, -0.07, 0.56, 0.4)$
